# mHealth Apps for Hypertension Self-Management: Interview Study Among Patient-Users

**DOI:** 10.2196/56162

**Published:** 2024-09-27

**Authors:** Felix Muehlensiepen, Dunja Bruch, Frances Seifert, Eileen Wengemuth, Martin Heinze, Sebastian Spethmann, Susann May

**Affiliations:** 1 Center for Health Services Research Faculty of Health Sciences Brandenburg Medical School Theodor Fontane Rüdersdorf Germany; 2 Department of Cardiovascular Surgery, Heart Center Brandenburg Faculty of Health Sciences Brandenburg Medical School Theodor Fontane Bernau Germany; 3 AGEIS laboratory Université Grenoble-Alpes Grenoble France; 4 University Clinic for Psychiatry and Psychotherapy Brandenburg Medical School Immanuel Hospital Rüdersdorf Rüdersdorf Germany; 5 Department of Cardiology, Angiology and Intensive Care Medicine Deutsches Herzzentrum der Charité Berlin Germany; 6 Charité – Universitätsmedizin Berlin, corporate member of Freie Universität Berlin and Humboldt-Universität zu Berlin Berlin Germany

**Keywords:** hypertension, mobile health, mHealth apps, digital health, patient perspective, qualitative study, cardiology

## Abstract

**Background:**

Hypertension is a major risk factor for cardiovascular disease, affecting over a billion people worldwide. Mobile health (mHealth) apps have emerged as effective tools for managing hypertension, offering capabilities for monitoring blood pressure, fostering lifestyle changes, and improving treatment adherence.

**Objective:**

This study aimed to explore patient-users’ perspectives on the hypertension care mHealth app Hypertension.APP, focusing on its accessibility, expected benefits, potential risks, and role in hypertension management in Germany.

**Methods:**

A qualitative study was conducted involving semistructured interviews with 20 patient-users of a hypertension care mHealth app, Hypertension.APP. Participants were recruited between January and June 2023 using purposive sampling. Verbatim transcripts were analyzed using qualitative content analysis.

**Results:**

Participants primarily discovered the app independently, driven by recent hypertension diagnoses and insufficient information from health care professionals regarding effective self-management strategies for their blood pressure. They valued the app for its continuous monitoring and feedback capabilities, aiding in understanding their condition and making lifestyle adjustments. Risks were perceived as minimal, mainly concerning data privacy and potential overreliance on the app. The app became integral to patient-users’ hypertension management by offering consistent information and support. The integration into formal health care was limited, as patient-users felt that health care professionals did not accept the use of the technology or might have even felt intimidated to use it.

**Conclusions:**

Among the sample studied, mHealth apps like Hypertension.APP were valued for their continuous monitoring and educational content, aiding in hypertension management. The findings suggest potential benefits of mHealth apps for effective hypertension care among patients who are health- and digitally literate as well as self-effective. There is a critical need for better integration of these apps into routine health care practices, as perceived by the app users. Given the small and specific sample of this qualitative study, further quantitative research with a broader and more varied participant group is necessary to validate these findings.

**Trial Registration:**

Deutsches Register Klinischer Studien DRKS00029761; https://tinyurl.com/r33ru22s

## Introduction

Despite effective guideline-based prevention and treatment options, cardiovascular diseases remain the leading cause of death worldwide [1]. Arterial hypertension is one of the most important risk factors for cardiovascular disease, affecting over 1 billion people worldwide [2]. In addition to genetic predisposition, the development of high blood pressure and the resulting cardiovascular outcomes are strongly influenced by individual factors, such as lifestyle and health literacy [3], as well as by local access to health care and its quality [4].

The recent increase in digital health technologies has opened new possibilities for the prevention and management of hypertension [5]. This surge includes mobile health (mHealth) applications, wearable devices, and other digital tools that empower individuals to monitor their blood pressure, track lifestyle changes, and set health goals. These developments offer health care professionals (HCPs) innovative methods to improve patient care and foster healthier living habits [6]. Among these, mHealth apps have shown particular effectiveness in treating hypertension [7].

Despite the significant potential of mHealth apps in hypertension care, their integration into routine hypertension care in Germany is sporadic. This hesitance can be attributed to factors that are well-recognized in other medical fields. These include a lack of awareness of the benefits of mHealth apps [8], an absence of reimbursement models for their integration into health care services [9], and interoperability difficulties with existing systems [10].

However, this study did not focus on these barriers. Instead, we sought to understand the perspectives and experiences of individuals with hypertension who actively use mHealth, specifically, Hypertension.APP, to manage their condition. This user-centered approach could provide insights into the practicalities, determinants, and challenges faced by patients, thereby informing strategies to optimize mHealth app usage in hypertension care.

Thus, the aim of this qualitative study was to explore user experiences with an mHealth app in hypertension care, focusing on app accessibility, expected benefits and perceived risks, and its role in hypertension care in Germany.

## Methods

### Study Design

We conducted a qualitative interview study on the perspectives of patient-users of an mHealth app for hypertension care. This study is part of the digital preventive measures for arterial hypertension (DiPaH) - project [11], which examines the structural and individual factors of patients and stakeholders in the use of digital preventive measures in patients with arterial hypertension in Germany.

### mHealth App: Hypertension.APP

**Figure 1:**
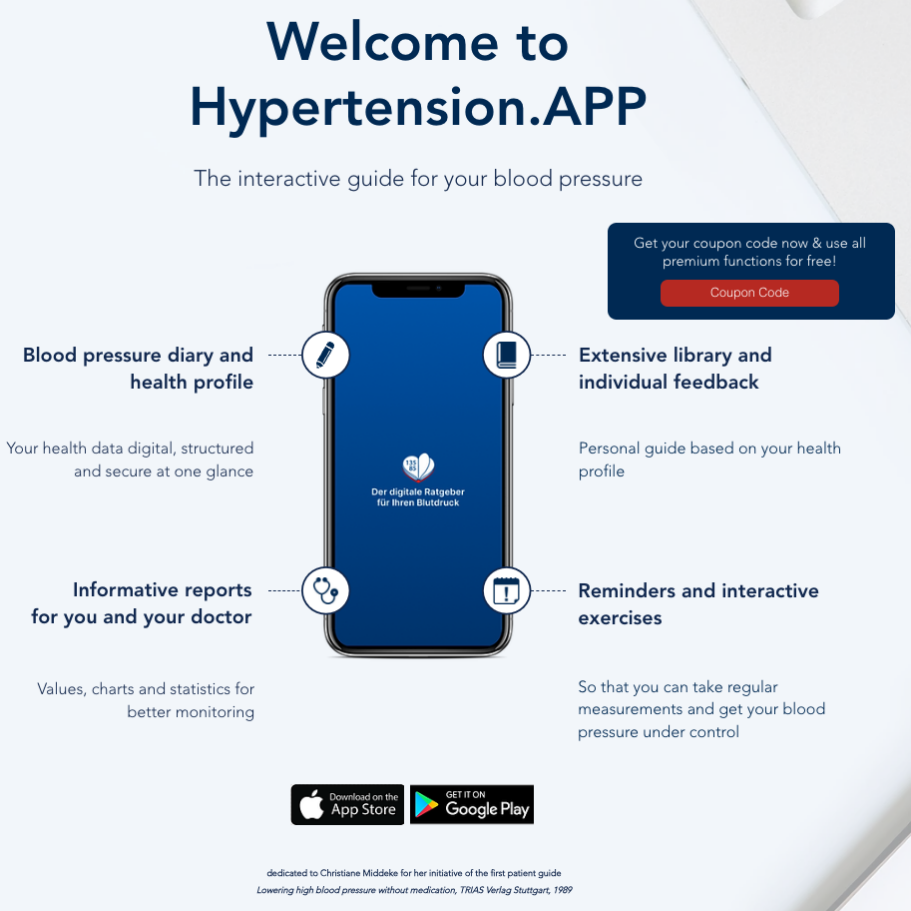
Visualization of the Hypertension.APP and its functions [12].

The app (Hypertension.APP) which patient-users were interviewed in this study was selected as an example according to expert recommendations (Prof Dr Martin Middeke and Prof Dr med Miklós Illyés). The Hypertension.APP is developed and maintained by the Hypertension Care UG (Ingolstadt) [12]. The application has been downloaded in app stores nearly 10,000 times as of May 2024. It includes a diary function to document blood pressure data, a personalized guide that provides information based on the user’s health profile, an export function to generate reports for medical consultation, a reminder function, and interactive exercises. The Hypertension.APP and its functions are shown in Figure 1 [12]. The app is freely accessible in various app stores. There is also a purchasable premium version which includes additional features.

### Recruitment

Patient-users were invited to participate in the study through the Hypertension.APP. A study invitation for the DiPaH study was placed in the app which led users to an online questionnaire survey and an online form for entering contact details for the interviews. The study team contacted people who had entered their contact details within 14 days and an interview appointment was arranged. Participants were selected using purposive sampling with the aim to include heterogeneous app users [13]. The inclusion criteria were age 18 years or more, use of the app in the last 2 years, and arterial hypertension. A further inclusion criterion was interest in and willingness to participate in the interviews. We chose to conduct 20 interviews as this number has proven to be adequate in previous studies to reach theoretical saturation in the exploration of digital approaches [14-16].

### Data Collection

A preliminary semistructured interview guide was drafted by a multiprofessional team (FM, SM, EW, DB, and SS). The semistructured interview guide consisted of open-ended questions that explored participants’ motifs to use the Hypertension.APP, its usability, and the advantages and disadvantages of the app, as well as its impact on personal hypertension care. Sample interview questions included: (1) “Please remember. How did you come to use this app?” (2) “Please tell me, what do you think of the app?” (3) “What role does the app play in your hypertension care?” In addition, sociodemographics and health-related data were collected, including profession, sex, age, location (rural or urban), federal state, education, technical devices, blood pressure values, antihypertensive drugs, time since diagnosis, comorbidities, and tobacco use. To test the applicability of this guide, we conducted 3 pilot interviews (1 to 3). The interview guide (Multimedia Appendix 1) proved to be appropriate, so the 3 pilot interviews were included in the analysis. All interviews were conducted by an experienced qualitative researcher (FM) by telephone, between January and June 2023. The interviews were recorded and transcribed. Data collection was continued until the aim of 20 interviews was achieved. Consistent with the planned number of interviews, no substantial new findings emerged, and saturation of content was reached [17].

### Data Analysis

Data were analyzed in parallel with the data collection by 2 experienced qualitative researchers (FM and SM). Analysis was based on Kuckartz’s structured qualitative content analysis [18] using MAXQDA Analytics Pro 2022 (version 22.1.0; Verbi GmbH). After transcription of the audio material, the analysis began with the screening of the interview texts, whereupon the interviews were coded. Relevant text passages from the interview materials were coded according to a deductive-inductive procedure. The main categories were developed deductively based on the research questions of the DiPaH study and subcategories were formed inductively from the codes. Consensus discussions were held continuously in the research group until a common understanding of all the emerging categories was achieved. After data collection was concluded, the 2 researchers (FM and SM) separately applied the established category system to analyze the complete dataset, ensuring that the process could be traced and replicated. For the presentation of the results, representative quotes from the transcripts were selected, translated into English, and included into the manuscript.

### Ethical Considerations

The study was approved by the ethics committee of the Brandenburg Medical School Theodor Fontane (E-02-20220620). The study was registered as part of the DiPaH project at German Clinical Trials Register (DRKS00029761). Primary data collection was conducted in compliance with the current data protection regulations of the General Data Protection Regulation and the Helsinki declaration. Participants received extensive study information and provided written informed consent. Data were pseudonymized before analysis. No conclusions can be drawn about individuals in the presentation of results. Participants received €30.00 (US $33.07) as an incentive and compensation for their participation. Patients were not involved in designing this study. The manuscript has been compiled in accordance with the COREQ (Consolidated Criteria for Reporting Qualitative Research; Multimedia Appendix 2) [19]. All methods were performed in accordance with relevant guidelines and regulations.

## Results

### Participant Characteristics

A total of 20 interviews with 20 participants were conducted and were included in the analysis. The mean duration of the interviews was 26 (10-40) minutes. The mean age of the participants was 56 (range 29-80) years. In total, 10 females and 10 males were interviewed. Out of 20, 13 participants were located in an urban area (>20,000 inhabitants), whereas 7 were located in a rural area (<20,000 inhabitants). The systolic blood pressure level most frequently reported was 130-139 mm Hg and diastolic blood pressure was <80 mm Hg. Most of the participants (16/20) were on blood pressure-lowering medication. Half of the participants were diagnosed with hypertension 1-5 years ago. None of the participants were currently using tobacco, while 7 had used tobacco earlier. All participants of this study used the free version of the app. Table 1 shows further information on participant characteristics.

**Table 1:** Detailed characteristics of the study participants.

Number	Age (years)		Sex		Location		Education		Blood pressure (mm Hg)				Antihypertensive drugs		Diagnosis (years)		Other Disease		Tobacco use	
									Systolic		Diastolic									
1	80		Male		Rural		University		120-129		<80		Yes		>10		Cardiovascular surgery		No	
2	59		Male		Urban		University		130-139		90-99		Yes		5-10		Elevated cholesterol levels		No	
3	69		Female		Urban		University		140-159		<80		No		1-5		Cardiovascular surgery		In the past	
4	42		Male		Urban		Intermediate		120-129		<80		Yes		1-5		None		No	
5	65		Male		Rural		Highschool		140-159		90-99		Yes		1-5		None		No	
6	29		Female		Rural		University		140-159		80-84		Yes		1-5		None		No	
7	64		Female		Rural		Intermediate		120-129		<80		No		1-5		Elevated cholesterol levels		In the past	
8	48		Female		Urban		Highschool		<120		<80		Yes		>10		Heart attack or stroke, elevated cholesterol levels		In the past	
9	48		Female		Urban		University		130-139		<80		Yes		<1		None		No	
10	64		Female		Urban		University		120-129		<80		Yes		1-5		None		No	
11	66		Female		Urban		University		130-139		80-84		No		1-5		Elevated cholesterol levels		No	
12	53		Female		Rural		University		120-129		85-89		Yes		5-10		None		No	
13	42		Male		Rural		University		130-139		80-84		Yes		>10		Elevated cholesterol levels		No	
14	54		Female		Urban		University		130-139		85-89		Yes		<1		Heart attack or stroke, cardiovascular surgery		In the past	
15	35		Male		Urban		Highschool		140-159		100-109		No		1-5		None		In the past	
16	53		Male		Urban		Highschool		130-139		80-84		Yes		1-5		None		No	
17	58		Male		Urban		University		120-129		85-89		Yes		1-5		Heart attack or stroke, cardiovascular surgery, elevated cholesterol levels		No	
18	49		Male		Urban		Highschool		120-129		90-99		Yes		>10		Elevated cholesterol levels, chronic kidney disease		In the past	
19	80		Male		Rural		Highschool		130-139		80-84		Yes		>10		None		In the past	
20	53		Female		Urban		Highschool		130-139		80-84		Yes		>10		None		No	

### Themes

A total of 4 key themes were explored in the analysis: (1) access to mHealth apps in hypertension care, (2) expected benefits of using mHealth apps in hypertension care, (3) potential risks of using apps in hypertension care, and (4) role of the app in hypertension care routines. Table 2 contains the details of the final coding tree.

**Table 2:** Coding tree of the qualitative content analysis.

Themes and categories			Code (examples)
**Access to mHealth^a^ apps in hypertension care**			
	Discovery and adoption	Self-discovery, third-party sources, self-efficacy, and eHealth literacy	
	Motivation	Convenience, enhancing knowledge, aid in disease management, tackling the disease, control over the disease, and advantages over paper documentation	
	Downloading and installation	User experience, support, technical challenges, and ease of navigation	
**Expected benefits of using mHealth apps**			
	Monitoring and documentation	Long-term monitoring, detection of blood pressure trends, and comparison with analog documentation	
	Feedback and information	Immediate explanations and feedback, personalized health advice, and lifestyle adjustments based on app feedback	
	Independence in illness management	Self-management of health conditions, blood pressure control, enhanced self-awareness, and adherence	
**Potential risks of mHealth in hypertension care**			
	Data protection and privacy	Concerns about data misuse and security breach fears	
	App usage	Risks of over-reliance on the app and misinterpretation of data	
**Role of mHealth app in hypertension care routines**			
	Integration in self-management	Daily routine integration, personification of the app, and emotional support	
	Integration of mHealth app in formal health care	Use of app information in medical consultations; reactions of medical staff to the app; health care provider skepticism, and gap between app use and medical practice	
	User trust and reliability	Confidence in the app’s information and the app as a decision support tool	

^a^mHealth: mobile health.

### Access to mHealth Apps in Hypertension Care

The participants reported that the diagnosis of high blood pressure was the initial reason for using the app:

I actually came across it by chance. Because two years ago I was diagnosed with high blood pressure. And the doctor said that I should always measure my blood pressure and keep an eye on it. So I had a look to see if there were any little aids available. In the app store, right? Then I came across the app. It was also well-rated, right? And, yes, I downloaded it and, yes, it was also easy to use. And, I have actually been using the app ever since. And I always record my blood pressure. Regularly, right? The measurements. So that I can keep an eye on how it's developing. Does it go too high at first or is it too low?

Interview_4, pos. 7

The participants recounted that they had discovered the app mostly on their own. They either searched directly for an app on hypertension in the app store or they searched for further information on hypertension and then became aware of the app through relevant websites.

Interviewer: Do you remember how you came to use the app?

Participant: I don't remember exactly. I looked at various apps in the app store and tried out which ones I got on well with, and then at some point I found the hypertension app, because it not only records, but also provides information about it.

Interviewer: And you did it all on your own, without any advice from a doctor or anything like that?

Participant: No, my doctor never said anything about the app. I looked for it myself.

Interview_12, pos. 6-9

The participants recalled their dissatisfaction with the information provided by their treating HCPs concerning effective self-management strategies for blood pressure and undertook their own research endeavors. They independently searched for information on their condition to positively influence it through lifestyle changes. In doing so, they differentiated reliable sources from others.

I was recently diagnosed with high blood pressure at the beginning of the year which is still relatively recent, and I didn't really get any useful information from my GP. I felt a bit ill-advised or in bad hands, so I did some research on the Internet to find reliable sources and then came across the German Heart Foundation, where I ordered a magazine. It's called “Bluthochdruck, Herz und Gefäß schützen” [Engl.: High blood pressure, protecting the heart and blood vessels] and in this magazine this app was featured or advertised and that's how I came across this app.”

Interview_9, pos. 11

The patients experienced no problems in downloading or installing the app. Overall, the methods by which the participants accessed the app demonstrated high levels of self-efficacy which was also reflected in the expected benefits of using the app.

### Expected Benefits of Using Apps in Hypertension Care

The main motivation for using the app was the desire to save and continuously monitor blood pressure values on a long-term basis and to provide a clear overview to organize one’s own lifestyle and make adjustments to it. This was in contrast to the selective blood pressure recording during doctor visits.

(…) in order to monitor my values and then not only to selectively obtain values at doctor’s visits and get a snapshot or a 24-hour recording, but to actually be able to read certain trends and then act accordingly in everyday life.

Interview_16, pos. 77

From the participants’ perspective, one advantage of documentation through the app over other types of documentation is that patient-users receive immediate explanations and feedback on their values.

Yes, ultimately because I also get information and don't just read, “This is this number”, but what it means and what I can do.

Interview_12, Pos. 17

The app users relate this feedback directly to their lifestyle and adapt it if necessary. The app provides a basis for decisions regarding behavioral changes.

And I look how it goes and how my blood pressure level developed and draw my conclusions from that. For example, I realize that stress is actually a factor that increases my blood pressure. I can see this quite clearly because I know what I have done and when. And yes, you cannot always avoid stress, but you can try to limit it. So this feedback is really helpful.

Interview_17, Pos. 61

Compared with analog and paper-based documentation types, the overview and convenience of documentation through the app were emphasized by the participants.

Well, in the past it was important to me that I always wrote everything down by hand. So at the very beginning, yes, there was a little booklet from the blood pressure machine. But then the booklet was full. I just didn’t enjoy writing everything down by hand. And that immediately convinced me to enter this data. I do a lot on my smartphone. And yes, and that I can also see immediately whether I have hypertension or whether it’s the same and so on. So the values that I enter also give me immediate information about the status of things. So that convinced me immediately.

Interview_11, pos. 20

The participants emphasized the importance of managing their illnesses independently. They reported that the app effectively helped them achieve this objective, particularly by controlling their blood pressure.

Because I consider it important to have my blood pressure under control given my state of health.

Interview_1, Pos. 105

One app user also reported that the app enabled him to lead an independent life and that independent app-supported monitoring helped him visit the doctor less often.

Because I lead a very unconventional life. It is imperative that I need as little medical help as possible. That is why I have to get a tight grip on my incipient illnesses – after all, I am 80 years old. I have to be in control of the situation.

Interview_19, Pos. 19

### Potential Risks of mHealth in Hypertension Care

In addition to the perceived benefits, participants were also asked about potential risks of using the app. However, most participants did not perceive any risk when using the app. Some participants addressed the general risk of misuse of data by third parties:

Well, I do see a risk that you enter health data here, of course. This is just the data protection risk. But not specifically in this app, it’s a general story. I think the app is relatively well-secured. And yes, the data can sometimes be hacked, okay. That always raises the question of who uses this data and for what?

Interview_16, pos. 55

Furthermore, the participants reported a risk of excessive use of the app.

Interviewer: Do you see any risks when using the app?

Participant: Well, not for me. Maybe that you get too attached to the app. But that’s always the case with things like this. But I tend to forget that.

Interview_10, pos. 72-73

### Role of an mHealth App in Hypertension Care Routines

In the interviews, the participants described the app as an essential element in the self-management of their own hypertension care routines. In contrast to conventional hypertension care, continuous availability of support and information was emphasized in the interviews. In some cases, the app was even personified and its use was described as a treatment.

Well, all these things combined make for perfect prevention or even treatment. It is actually already a form of treatment, yes. Because look, how often do you visit your doctor? To my general practitioner maybe twice a year. So they always tell you the same. And that’s it, yes. But I need this support on a daily basis, yes. It [the app] is like a kind of companion, like a sparring partner basically, yes. And exactly, the app is my sparring partner because it also communicates with me.

Interview_11, pos. 30

The app provides information to understand one’s condition. Participants described that the app supports communication with the medical staff and enables patient-users to express their needs.

And as I said, the most important thing for me was to be taken seriously by the doctor. And when you engage more with your own illness, then you can, well, argue better.

Interview_5, Pos. 53

In addition, the interview participants reported that they had confidence in the app and regarded the information presented as reliable. Simultaneously, the significance that the user attaches to the app’s information is not acknowledged in the doctor’s office.

Well, I must say, I trust the app and have already read the information, which is kept relatively concise, from the library. And yes, because I trust the app, as I have already mentioned, I take seriously what it shows me, to put it bluntly. And so, for me, that is trustworthy information. (…) And with this mild or slight diastolic hypertension, it then says, “Continue taking your blood pressure medication and consult a doctor to further optimize the medication or something in that direction.” Exactly, so I find it very valuable information. But I had the impression which might also be due to the individual doctor that the lady didn't take my concern to discuss the medication again very seriously.

Interview_13, Pos. 50

Similarly, another user suggested that the treating doctor might feel intimidated by the app or the information provided. The app is currently not integrated into the user’s medical hypertension care.

So, in the general practitioner's office, it seems like the app has not really caught on there, I feel. If I were in the place of the general practitioner, I would really take it seriously. Of course, it is possible that the general practitioner might feel threatened by it. I don't know. (...) If I were in her shoes, I would have been more interested. She did say, “Oh that's great. And good that you're doing it.” But she didn't say, “Show me the app.” or anything like that...

Interview_11, Pos. 34

## Discussion

### Principal Findings

This qualitative study is the first to explore user experiences with an mHealth app in hypertension care, focusing on app accessibility, expected benefits, potential risks, and its role in hypertension care in Germany. The study showed that patient-users of the Hypertension.APP typically discovered the app independently, driven by a combination of a recent high blood pressure diagnosis and a lack of satisfactory information from their HCP. These patient-users particularly appreciated the app’s capabilities for continuous monitoring and documentation of blood pressure, as it offered valuable feedback and explanations, aiding them in understanding their condition and making informed lifestyle adjustments. While acknowledging these benefits, patient-users also noted potential risks concerning data privacy and the possibility of becoming overly reliant on the app, although they assigned relatively low importance to these concerns. The app has become an essential part of users’ disease management routines, providing consistent support and information that contrasts with low-frequency traditional doctor visits. Regarding the disappointment of app users, the integration of the app into formal health care settings was limited, with some patient-users sensing a lack of acceptance or even intimidation from the HCP toward the technology.

### Comparison With Previous Work

Our results clearly indicate that mHealth can be a valuable tool for self-management of arterial hypertension. This finding is in concordance with existing research, underscoring the substantial potential and effectiveness of digital health [5,6], particularly mHealth [7], not only in hypertension care but also in other medical areas [14-16].

While in other medical domains the use of digital technologies is associated with depersonalization or dehumanization of health care [14,20], in this study, participants personified the app or considered it a “sparring partner.” We hypothesize that providing personalized information enhances user motivation and commitment to app usage, and therefore recommend this for the development of digital health applications.

From a socio-technological perspective, our findings resonate with Bruno Latour's actor-network theory [21]. This theory characterizes an “actant” as an entity that either acts or is granted action by others, without necessarily attributing specific motivations to human actors or humans in general. In this framework, any entity can be an actant as long as it is acknowledged as the origin of an action. In the realm of hypertension management, our data position the app as a pivotal actant within the actor network. Remarkably, for some patient-users, the app assumed a more influential role than that of the health care professionals involved, underscoring its significance in this domain.

Notably, the participants expressed dissatisfaction with the lack of integration of mHealth into standard hypertension care. They advocated its inclusion in formal hypertension practices, stressing the need for HCPs to recognize and leverage these digital health tools. Why do German HCPs have reservations about mHealth in hypertension care? The reluctance of German HCPs toward mHealth in hypertension management is a multifaceted issue, as explored in a separate module of the DiPaH study [11]. HCPs reported doubts regarding the validity of patient-generated data, data privacy concerns, and additional efforts including training and interoperability. These factors, along with the absence of incentives and insufficient incorporation into the health care system’s reimbursement frameworks, are major hindrances to the full exploitation of mHealth capabilities, despite the advantages acknowledged by patients. This contrasts with other chronic conditions, such as rheumatoid arthritis, where HCPs acknowledge the value of mHealth in facilitating shared decision-making and adopting a treat-to-target approach [15].

### Implications

It appears critical that patient-users of mHealth applications, especially those managing chronic diseases, receive serious attention and support from their HCPs. Therefore, potential users and stakeholders should be involved in the development of digital health applications. This was the case for the development of Hypertension.APP. However, there is currently a significant shortfall in the information resources available to HCPs which is crucial as there is evidence that HCPs’ knowledge of telemedicine is closely intertwined with their actual use of telemedicine services [22]. Low-threshold training opportunities for HCP are highly needed and should be supported by professional organizations involved in hypertension care.

Looking ahead, the narratives from our interview participants suggest that they possessed a high level of self-efficacy and eHealth literacy, as well as a higher income and educational background. This observation raises critical questions about accessibility and equity: who is able to use these mHealth services effectively, and who is not? Based on interviews alone, we can only speculate; however, a more comprehensive analysis combining interview and questionnaire data would offer valuable insight into these dynamics. It could provide a clearer picture of the demographic profile of current patient-users of mHealth in hypertension care, and potentially identify groups that are currently underserved or unable to benefit from this digital health technology.

This knowledge would be instrumental in developing strategies to make mHealth more accessible and effective for a broader range of patients, particularly those with chronic conditions, such as hypertension.

### Strengths and Limitations

While our study offers valuable insights, it is essential to acknowledge its limitations. First, the inclusion of solely patient-users, particularly limited to one specific app may introduce selection bias, potentially limiting the generalizability of our findings. In addition, the perspectives of treating HCP, particularly GPs and cardiologists, warrant exploration in a separate study to provide a comprehensive understanding of hypertension management. Specifically, investigating the optimal integration of app data into the local electronic medical record and the workflow for HCPs is imperative, as this aspect could significantly influence their acceptance of digital hypertension care.

In addition, our study may be susceptible to self-selection bias and social desirability bias, exacerbated by the provision of study incentives. Furthermore, the demographic composition of interview participants, predominantly consisting of highly educated nonsmokers, fails to fully represent the diverse population living with hypertension. The absence of reported issues during the download and installation of the app suggests that our sample might also possess higher levels of self-efficacy, eHealth literacy, and technical skills compared with the general population. This indicates a potential overestimation of these attributes in the broader community of hypertensive patients. Most participants had controlled blood pressure levels, with only a minority having systolic blood pressure >139 mm Hg and some not on medication. This may also have influenced the HCPs perceived need for mHealth based blood pressure monitoring.

Furthermore, it is crucial to acknowledge that the app used in our study lacks certification as a digital therapeutic (“Digitale Gesundheitsanwendung” or DiGA), limiting its integration into standard health care systems and reimbursement schemes.

However, despite these limitations, our study stands as the first exploration of user experiences with an mHealth app for hypertension care in Germany. By closely examining the realities of care and patient perspectives, our findings offer valuable insights into the potential of digital self-management in hypertension care and highlight areas where further improvement is needed.

### Conclusion

Among the sample studied, mHealth apps like Hypertension.APP were highly valued for their continuous monitoring and educational content, which aids in hypertension management. The findings highlight the potential of mHealth apps for effective hypertension care among patients who are health- and digitally-literate, as well as self-effective. However, there is a critical need for better integration of these apps into routine health care practices, as perceived by the users. This includes addressing the skepticism and lack of acceptance among HCP, ensuring that they receive the necessary training and resources to effectively use these tools. Further research should also focus on the accessibility and effectiveness of mHealth apps across diverse patient populations to promote equitable health benefits.

## Appendix

Multimedia Appendix 1 Interview guide.

Multimedia Appendix 2 COREQ (Consolidated Criteria for Reporting Qualitative Research) checklist.

## Abbreviations

COREQ: Consolidated Criteria for Reporting Qualitative Research
DiPaH: digital preventive measures for arterial hypertension
HCP: health care professional
mHealth: mobile health
