# Supplementary material for: mHealth Apps for Hypertension Self-Management: Interview Study Among Patient-Users
Source: JMIR Form Res. 2024 Sep 27;8:e56162. doi: 10.2196/56162 (PMC11470216; doi:10.2196/56162)
Supplement: Multimedia Appendix 1 [file formative_v8i1e56162_app1.pdf]

**Interview Guide: Digital preventive measures for arterial hypertension - Module 2b**

<u>Research questions</u>

What motivates participants to use the *Hypertension.APP*?
Which treatment paths were used and what hurdles had to be overcome?
What advantages and disadvantages were experienced in comparison to purely analog measures?
How continuously was the app used?
To what extent is the use of a hypertension app included in the interaction with the treating physician?
Is the medical treatment adapted through its use?
How can the data be used for GP care (interfaces)?
What are the characteristics of (digital) health literacy?

| Key question/ narrative impulse | Check aspects | Concretizing questions | Maintenance and control issues |
|---|---|---|---|
| You have been using the *Hypertension.App* for some time.<br><br>Please remember. How did you come to use this app? | Warm-up<br><br>Motivation<br><br>General expectations<br><br>Expected change<br><br>Setting | When was that? When did you start using the app?<br><br>How did you find out about the app?<br><br>Who (healthcare provider) have you spoken to about the app?<br><br>Why did you decide to use the app?<br><br>What did you think would change?<br><br>Did you have any concerns?<br><br>How easy or difficult is it for you to familiarize yourself with new technologies?<br><br>Have you ever used technology or digitalization in your healthcare? | Can you tell us more about this?<br><br>And then?<br>How was that for you?<br><br>How do you see that? Can you please go into this in more detail?<br><br>Could you please give an example? |

| | | | |
|---|---|---|---|
| Please tell me, what do you think of the app? | User experience<br><br>Usage behavior<br><br>User-friendliness<br><br>Handling<br><br>Visual, haptic aspects<br><br>Comparison<br><br>Expectations vs. reality<br><br>Expenditure | When do you use the app?<br><br>How long does it take?<br><br>How often do you use the app? (Does the app change your everyday life?)<br><br>What works well?<br><br>Is there anything that doesn't work so well? If so, what?<br><br>Is the app easy to use?<br><br>How was the download / installation? Did everything work?<br><br>And what else? Was there anything or anyone who made it easier for you to use the app or to get started? (Family, specialist staff, etc.)<br><br>Do you understand the instructions?<br><br>Do you always know what to do?<br><br>If you think back to your original expectations, does the app meet your expectations?<br><br>Do you see any risks in using the app?<br><br>Is there anything you would change about the app? | What do you mean in concrete terms?<br><br>Can you tell us more about this?<br><br>And then?<br><br>How was that for you?<br><br>How do you see that?<br><br>Can you please go into this in more detail?<br><br>Could you please give an example? |

| | | | |
|---|---|---|---|
| What role does the app play in your hypertension care? | Care process<br><br>Interview with a doctor<br><br>Personal attitude | Do the doctors look at your values documented via the app? Do the doctors address this in the consultation?<br><br>Which doctors? Cardiologist? Also GPs ? Others?<br><br>Have you ever spoken to non-medical staff about your documented values?<br><br>Have you ever been contacted outside of consultation hours regarding your documented values?<br><br>Are there analog offers that could support the app?<br>➔ Further contact requests: HA, pharmacy, etc.<br><br>Does the app affect your state of health?<br>If yes, in what way? / If no, why not?<br><br>We are slowly coming to the end of our interview:<br><br>If you now take look at your treatment with and without the app, are there any differences? Has anything changed?<br><br>Does the app influence how you experience your illness?<br><br>Does the app influence how you deal with health or illness information?<br><br>Will you continue to use the app? For what reasons? | What do you mean in concrete terms?<br>Can you tell us more about this?<br>Can you tell us more about this?<br>And then?<br>How was that for you?<br>How do you see that?<br>Can you please go into this in more detail?<br>Could you please give an example? |
